# Radiation-Induced Contrast Enhancement After Proton Therapy for Paediatric Brain Tumours: Association with Variable Relative Biological Effectiveness and Linear Energy Transfer

**DOI:** 10.3390/cancers18193243

**Published:** 2026-10-08

**Authors:** Julian Brack, Valon Gllareva, Bastian von Nettelbladt, Eva Meixner, Line Hoeltgen, Laila Koenig, Hanna Waldsperger, Katharina Kozyra, Habiba Sallem, Alexander Neuholz, Thomas Tessonnier, Andrea Mairani, Jürgen Debus, Klaus Herfarth, Semi B. Harrabi

**Affiliations:** 1 Department of Radiation Oncology, Heidelberg University Hospital, 69120 Heidelberg, Germany; 2 Heidelberg Institute for Radiation Oncology (HIRO), 69120 Heidelberg, Germany; 3 Heidelberg Ion-Beam Therapy Center (HIT), Heidelberg University Hospital, 69120 Heidelberg, Germany; 4 Cantonal Hospital Frauenfeld, Spital Thurgau Ag, Pfaffholzstrasse 4, 8500 Frauenfeld, Switzerland; 5 National Center for Tumor Diseases (NCT), 69120 Heidelberg, Germany; 6 Clinical Cooperation Unit Radiation Oncology, German Cancer Research Center (DKFZ), 69120 Heidelberg, Germany; 7 Translational Radiation Oncology, German Cancer Research Center (DKFZ), 69120 Heidelberg, Germany; 8 Centro Nazionale di Adroterapia Oncologica (CNAO), 27100 Pavia, Italy; 9 German Cancer Consortium (DKTK), Partner Site, 69120 Heidelberg, Germany

**Keywords:** proton therapy, relative biological effectiveness, linear energy transfer, radiation-induced contrast enhancement, paediatric brain tumours, ependymoma, low-grade glioma

## Abstract

**Background/Objectives**: Proton therapy is widely used in paediatric neuro-oncology because of its favourable dose distribution and reduced exposure to radiation of healthy tissue. Nevertheless, current knowledge regarding radiation-induced contrast enhancements (RICE) is largely derived from adult proton therapy cohorts, while evidence in paediatric patients remains limited and the underlying biological mechanisms are still incompletely understood. This study investigated the incidence of RICE in paediatric patients with low-grade glioma (LGG) and ependymoma and evaluated potential associations with linear energy transfer (LET) and variable relative biological effectiveness (RBE)-weighted dose distributions. **Methods**: In this retrospective single-centre study, paediatric patients with LGG or ependymoma were identified from a prospectively maintained institutional registry patients treated with proton therapy at the Heidelberg Ion-Beam Therapy Centre (HIT) between 2010 and 2024. Follow-up magnetic resonance imaging examinations were systematically reviewed for RICE. Lesions were contoured in RayStation and assessed dosimetrically using a fixed RBE of 1.1 and three variable RBE models. LET-weighted dose metrics and Dose_(RBE)_ × LET products were compared between RICE regions and planning target volumes (PTVs). Overall survival and progression-free survival were estimated using Kaplan–Meier method. **Results**: Seventy-six patients met the inclusion criteria, including 33 patients with LGG and 43 with ependymoma. A total of 724 follow-up magnetic resonance imaging examinations were reviewed. RICE occurred in six patients (7.9%), exclusively in the ependymoma subgroup (14.0%). Higher prescribed radiation dose was significantly associated with RICE occurrence (*p* = 0.031). Across all variable RBE models, biologically weighted dose estimates and Dose_(RBE)_ × LET products were consistently elevated within RICE regions compared with corresponding PTV regions, particularly at low- and intermediate-dose levels. Five-year overall survival rates were 91.7% for LGG and 86.6% for ependymoma, while corresponding progression-free survival rates were 84.4% and 69.3%, respectively. **Conclusions**: Variable RBE models identified biologically relevant dose heterogeneities within RICE regions that were not adequately captured by the conventional fixed RBE approach. These findings suggest that LET-associated biological dose escalation may contribute to radiation-related brain tissue changes after proton therapy and support further investigation and prospective validation of LET- and RBE-informed normal-tissue risk assessment in paediatric neuro-oncology.

## Simple Summary

Children with brain tumours often survive for many years after treatment, making the prevention of long-term side effects an important goal of modern cancer care. Proton therapy can reduce radiation exposure to healthy tissues compared with conventional radiation techniques, but treatment-related changes in normal brain tissue may still occur. Some of these changes appear on magnetic resonance imaging as contrast-enhancing lesions and can be difficult to distinguish from tumour recurrence. In this study, we investigated the occurrence of these radiation-related imaging changes in children treated with proton therapy for low-grade glioma or ependymoma. We also explored whether biological characteristics of proton radiation, including variations in energy deposition within the brain, may contribute to their development. A better understanding of these mechanisms may help improve treatment planning and further reduce the risk of radiation-induced side effects in paediatric patients.

## 1. Introduction

Central nervous system tumours are among the most relevant malignancies in childhood, representing the most common solid tumours and one of the leading causes of cancer-related mortality in paediatric patients [1,2]. Low-grade gliomas and ependymomas constitute two important tumour entities within this group. While paediatric low-grade gliomas are generally associated with favorable long-term survival, particularly after complete surgical resection, ependymomas show a higher risk of local recurrence and frequently require adjuvant radiotherapy as part of multimodal treatment [3]. As many affected children become long-term survivors, the prevention of treatment-related late effects has become a central objective in modern paediatric neuro-oncology.

Radiotherapy plays an essential role in the treatment of paediatric brain tumours but is limited by the high sensitivity of the developing brain to ionizing radiation. Potential late effects include neurocognitive impairment, endocrine dysfunction, vascular injury, and secondary malignancies, all of which are particularly relevant in children because of their long expected post-treatment survival [4]. Proton therapy has therefore gained increasing importance in paediatric neuro-oncology. Owing to the characteristic Bragg peak, proton beams allow highly conformal dose deposition within the target volume while reducing the dose to surrounding healthy tissues compared with conventional photon radiotherapy [5,6]. This physical advantage is especially valuable when tumours are located close to radiosensitive brain structures.

Despite these dosimetric benefits, radiation-induced imaging changes may occasionally occur following proton beam therapy (PRT). One such finding is RICE, characterized by newly appearing contrast-enhancing lesions on follow-up magnetic resonance imaging within previously irradiated brain tissue [7]. These lesions are often asymptomatic and may regress spontaneously; however, they can resemble tumour progression or radionecrosis and therefore complicate radiological interpretation and clinical decision-making [7,8]. Reported rates of RICE differ between adult and paediatric cohorts, suggesting that age, vascular comorbidity, tissue regeneration, tumour location, and treatment-related factors may all influence their occurrence [9].

The biological mechanisms underlying these imaging changes remain incompletely understood. In clinical proton beam therapy, dose calculation is commonly based on a constant RBE of 1.1. This convention enables standardized treatment planning but does not account for spatial variations in biological effectiveness along the proton beam path [10,11]. Experimental and modeling studies indicate that RBE may increase in regions of elevated linear energy transfer, particularly near the distal fall-off of the Bragg peak [12,13]. Several variable RBE models, including those proposed by Wedenberg, McNamara, and Carabe, have been developed to incorporate linear energy transfer, dose per fraction, and tissue-specific radiosensitivity into biological dose estimation [14,15,16].

However, the clinical relevance of variable RBE remains controversial. While some studies suggest that linear energy transfer-associated biological dose escalation may contribute to radiation-induced brain injury, others emphasize the uncertainty and limited clinical validation of current models [17,18]. This uncertainty is particularly relevant in paediatric patients, in whom even subtle radiation-related tissue changes may have long-term consequences. Current normal tissue complication probability models are still largely based on physical dose parameters and may insufficiently reflect the biological heterogeneity of proton beam therapy [19,20]. Consequently, there is a need for clinical data linking radiation-induced imaging changes to linear energy transfer and variable RBE-based dose parameters.

The primary objective of this study was to investigate whether RICE preferentially occurs in regions characterized by elevated LET and increased variable RBE-weighted dose following proton therapy in paediatric patients with low-grade glioma and ependymoma. Secondary objectives were to determine the incidence and radiological characteristics of RICE and to describe long-term oncological outcomes. We hypothesized that RICE would preferentially develop in regions of increased biological dose that are not adequately represented by the conventional fixed RBE approach. By integrating systematic radiological follow-up with volumetric lesion assessment and biologically informed dosimetric analyses, this study aims to provide clinical evidence for the potential relevance of LET and variable RBE in radiation-induced imaging changes and to support the future development of biologically informed proton treatment planning.

## 2. Materials and Methods

### 2.1. Study Design and Patient Cohort

This retrospective single-centre cohort study analysed data from a prospectively maintained institutional registry comprising consecutive paediatric patients treated with proton beam therapy at the HIT between January 2010 and February 2024. Patients were eligible for the overall survival (OS) analysis if they were younger than 18 years at the start of irradiation, had a histologically confirmed diagnosis of LGG or ependymoma, and underwent primary cranial proton beam irradiation. For the radiological, dosimetric, and PFS analyses, an additional requirement of at least 12 months of radiological follow-up was applied. Among 820 paediatric patients treated with PRT during the study period, 418 received cranial irradiation. Of the 132 patients diagnosed with LGG or ependymoma, all were included in the OS analysis using mortality data obtained from the German death registry. Seventy-six patients fulfilled the additional radiological follow-up criterion and were included in the radiological, dosimetric, and PFS analyses. This cohort comprised 33 patients with LGG and 43 with ependymoma. The remaining 56 patients (23 with LGG and 33 with ependymoma) were included exclusively in the OS analysis. Further clinical and radiotherapeutic characteristics of the 76 patients, stratified by tumour entity, are presented in Appendix A (Table A1). LGG and ependymoma were selected as clinically relevant paediatric brain tumour entities in which long-term survival underscores the importance of assessing radiation-induced normal tissue effects.

### 2.2. Radiological Assessment

All available follow-up magnetic resonance imaging examinations were retrospectively reviewed using a standardized dual-reader approach, with at least one reviewer being board-certified in radiology or radiation oncology. The last available pre-treatment magnetic resonance imaging examination served as the reference scan. Follow-up examinations were assessed sequentially for newly occurring or progressive contrast-enhancing lesions. RICE was defined as newly appearing contrast enhancement located outside the gross tumour volume (GTV) but within the high-dose region of the corresponding proton treatment plan. In contrast, newly detected contrast enhancement within the GTV was considered indicative of treatment-related blood–brain barrier disruption or tumour-associated imaging changes rather than RICE. Native and contrast-enhanced T1-weighted, T2-weighted, and fluid-attenuated inversion recovery sequences were evaluated where available. In cases of diagnostic uncertainty, imaging findings were reviewed jointly until consensus was reached. The observation period was limited to 120 months after treatment to ensure comparable follow-up assessment.

### 2.3. Dosimetric and Biological Assessment

Detected RICE lesions were manually contoured in RayStation version 11B and spatially correlated with the original PTV. Dose distributions were assessed using the clinically applied constant RBE value of 1.1 and three variable RBE models: Wedenberg et al. (2013), McNamara et al. (2015), and Carabe et al. (2012) [14,15,16]. The variable RBE models were implemented using scripts imported into RayStation version 11B and applied to dose and LETd data. A photon reference α/β ratio of 2.0 Gy was assumed for brain tissue. The exact mathematical formulations and numerical coefficients implemented in the scripts are provided in Appendix B (Table A2). LET-weighted dose parameters and Dose_(RBE)_ × LET products were calculated for RICE regions and corresponding PTV at predefined dose-volume levels, including D99, D98, D95, mean dose, D50, D2, and D1. As an exploratory analysis, normal tissue complication probability (NTCP) values were estimated using the previously published model-based planning approach to compare conventional physical dose-based risk estimates with the observed occurrence of RICE [11,20].

### 2.4. Statistical Analysis

Statistical analyses were performed using IBM SPSS Statistics version 27.0.1. Continuous variables were summarized using medians and ranges, and categorical variables using absolute and relative frequencies. Non-normally distributed continuous variables were compared using the Mann–Whitney U test. Categorical variables were analysed using the chi-square test or Fisher’s exact test, as appropriate. Paired dosimetric comparisons between RICE regions and PTV were performed using the Wilcoxon signed-rank test. Overall survival and progression-free survival were estimated using the Kaplan–Meier method, and group comparisons were conducted with the log-rank test. An additional exploratory descriptive comparison of overall survival and tumour progression according to RICE status was performed in the 76 patients with sufficient radiological follow-up. Although overall survival was assessed in the extended cohort of 132 patients, this comparison was restricted to patients with a systematically assessed RICE status to minimize potential misclassification. Given the small number of RICE cases, no formal statistical comparison was undertaken. A two-sided *p*-value < 0.05 was considered statistically significant. Because of the limited number of RICE events, multivariable regression analysis was not performed to avoid model overfitting.

### 2.5. Ethics, Data Availability, and Use of Generative Artificial Intelligence

The study was approved by the Ethics Committee of the University of Heidelberg, Germany (approval number S-377/2018). Individual-level clinical imaging and treatment data are not publicly available because of data protection regulations and institutional ethical requirements. Anonymized derived datasets may be made available by the corresponding author upon reasonable request and subject to institutional and ethical approval. No publicly deposited large-scale dataset or accession number is associated with this study.

## 3. Results

### 3.1. Patient Characteristics and Follow-Up

**Figure 1:**
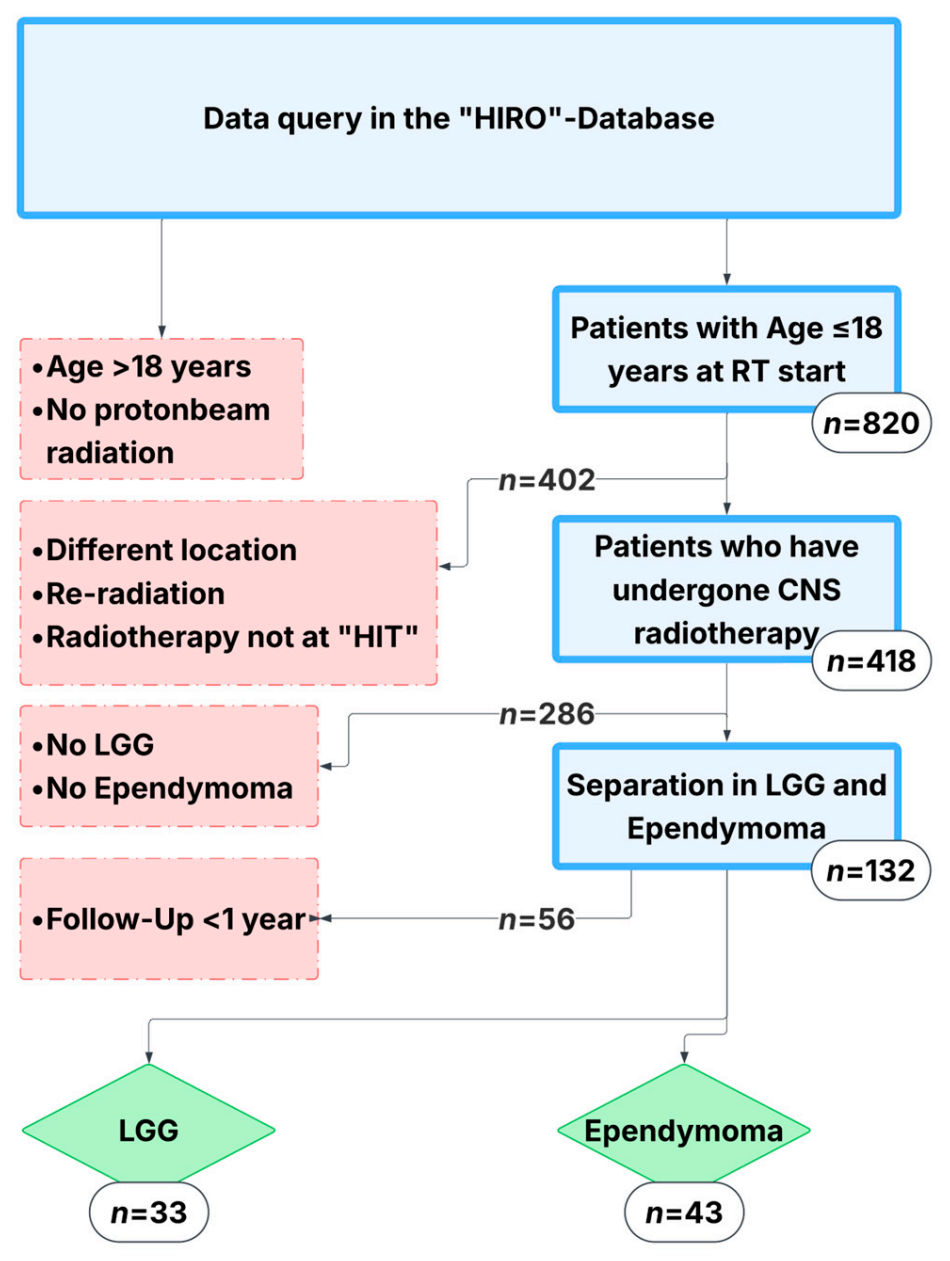
CONSORT diagram illustrating the patient selection workflow used in this project. Blue rectangles indicate the search parameters applied at each filtering step, red rectangles indicate the corresponding exclusion criteria, and green diamonds represent the final patient cohort included in the subsequent analyses.

The final radiological and dosimetric analysis cohort comprised 76 paediatric patients, including 33 patients with low-grade glioma and 43 patients with ependymoma. The cohort consisted of 43 male and 33 female patients. The median age at the start of PRT was 11 years in the low-grade glioma group and 5.6 years in the ependymoma group. In the low-grade glioma subgroup, 28 patients had World Health Organization (WHO) grade I tumours, four had grade II tumours, and one patient had low-grade glioma not otherwise specified. In the ependymoma subgroup, four patients had WHO grade II tumours and 39 patients had WHO grade III ependymomas. The median follow-up was 38.5 months, ranging from 13 to 145 months. The patient selection process is shown in Figure 1.

### 3.2. Survival and Progression Outcomes

For overall survival analysis, the extended cohort of 132 eligible patients was evaluated using mortality data obtained from the German death registry. Median overall survival was not reached in either tumour entity. The estimated 5- and 10-year overall survival rates were 91.7% and 84.0% for low-grade glioma and 86.6% and 75.5% for ependymoma, respectively. No statistically significant difference in overall survival was observed between both entities (*p* = 0.41).

Progression-free survival was analysed in the 76-patient cohort with available long-term data (*n* = 76). Five- and 10-year progression-free survival rates were 84.4% and 77.3% in patients with low-grade glioma and 69.3% and 63.0% in patients with ependymoma, respectively. Although progression-free survival tended to be lower in the ependymoma group, the difference did not reach statistical significance (*p* = 0.07). Tumour progression occurred in 17 of 76 patients (22.4%), including five patients with low-grade glioma (15.2%) and twelve patients with ependymoma (27.9%). Median time to progression was 27 months, with a range of 2 to 107 months. Male patients experienced significantly earlier progression than female patients, with median times to progression of 24.8 and 56.2 months, respectively (*p* = 0.031).

An exploratory descriptive comparison of oncological outcomes according to RICE status was performed in the 76 patients with available radiological follow-up. Among the six patients who developed RICE, no deaths and one tumour progression (16.7%) were recorded, compared with ten deaths (14.3%) and 16 progression events (22.9%) among the 70 patients without RICE. The median recorded observation times for the OS endpoint were 38 and 63 months in patients with and without RICE, respectively. Given the limited number of RICE cases, no formal statistical comparison was performed.

### 3.3. Treatment Characteristics

The median prescribed radiation dose across the entire cohort was 54 Gy, with a range of 48.6 to 68 Gy. Patients with low-grade glioma received a median dose of 54 Gy, whereas patients with ependymoma received a significantly higher median dose of 59.4 Gy (*p* < 0.001). Two-field irradiation was the most frequently used treatment approach in both groups, applied in 26 of 33 low-grade glioma patients and 28 of 43 ependymoma patients. PRT was predominantly administered after prior surgical intervention and represented second-line treatment in 60.6% of low-grade glioma patients and 72.1% of ependymoma patients.

### 3.4. Incidence and Morphological Characteristics of Radiation-Induced Contrast Enhancements

RICEs were detected in six of 76 patients, corresponding to an overall incidence of 7.9%. All affected patients belonged to the ependymoma subgroup, resulting in an incidence of 14.0% among patients with ependymoma, whereas no RICEs were observed in the low-grade glioma group. The exact 95% confidence intervals were 14.0% (6/43; 95% CI 5.3–27.9%) for ependymoma and 0% (0/33; 95% CI 0–10.6%) for low-grade glioma. The sex distribution among affected patients was balanced, with three male and three female patients.

Four patients showed small transient RICE lesions that were visible in only one follow-up magnetic resonance imaging examination. Two patients developed classic periventricular RICE with persistent contrast uptake over multiple follow-up examinations. All observed lesions demonstrated spontaneous regression over time.

A higher total prescribed radiation dose was significantly associated with the occurrence of RICE (*p* = 0.031). Patients with RICE had received a mean dose of 58.5 Gy, compared with 55.3 Gy in patients without such lesions. All six RICE cases occurred within the ependymoma subgroup, which received a significantly higher median prescribed dose than the low-grade glioma subgroup (59.4 vs. 54 Gy, *p* < 0.001). Within the ependymoma subgroup, RICE incidence varied according to the number of irradiation fields. RICE occurred in two of four patients (50.0%) treated with a single field, two of 28 patients (7.1%) treated with two fields, and two of 11 patients (18.2%) treated with three fields. Given the small number of RICE cases, these differences should be interpreted descriptively.

### 3.5. Dosimetric and Biological Characterization of Radiation-Induced Contrast Enhancement Regions

The median volume of contoured RICE lesions was 0.16 cm^3^ (0.01–0.68 cm^3^). In comparison, the median PTV was 167.61 cm^3^ (45.01–419.12 cm^3^). To complement the voxel-wise dosimetric analyses, exploratory NTCP estimates were calculated for the five patients with periventricular RICE using our previously published risk prediction model [11,20]. Predicted NTCP values ranged from 0.05 to 0.52, with a median of 0.12.

Mean Dose_(RBE)_ × LETd products were higher in RICE regions than in PTV at low- and intermediate-dose levels (D99–D50), whereas PTV values were higher at D2 and D1 (Table 1). Direct comparison of the underlying metrics showed higher mean LETd in RICE regions, particularly at D99 and D50. In contrast, conventional RBE-weighted dose (fixed RBE = 1.1) was nearly similar between RICE regions and PTV at D99 and lower in RICE regions at D50 to D1 (Table 2). Thus, elevated combined dose–LET metrics within RICE regions were not accompanied by a consistently higher conventional RBE-weighted dose. Given the small RICE lesion volumes, dose-volume metrics were evaluated from D99 to D1. Metrics below D1 were excluded because of their increasing sensitivity to contouring, image-registration, voxel-size, and local dose-gradient uncertainties.

To facilitate interpretation of the combined Dose_(RBE)_ × LET metric, Table 2 separately presents the mean RBE-weighted dose and LET values for the eight RICE regions and six corresponding PTV. LET was higher within RICE regions, particularly at low- and intermediate-dose levels, whereas the corresponding RBE-weighted dose values were generally similar to or lower than those observed in the PTV.

**Figure 2:**
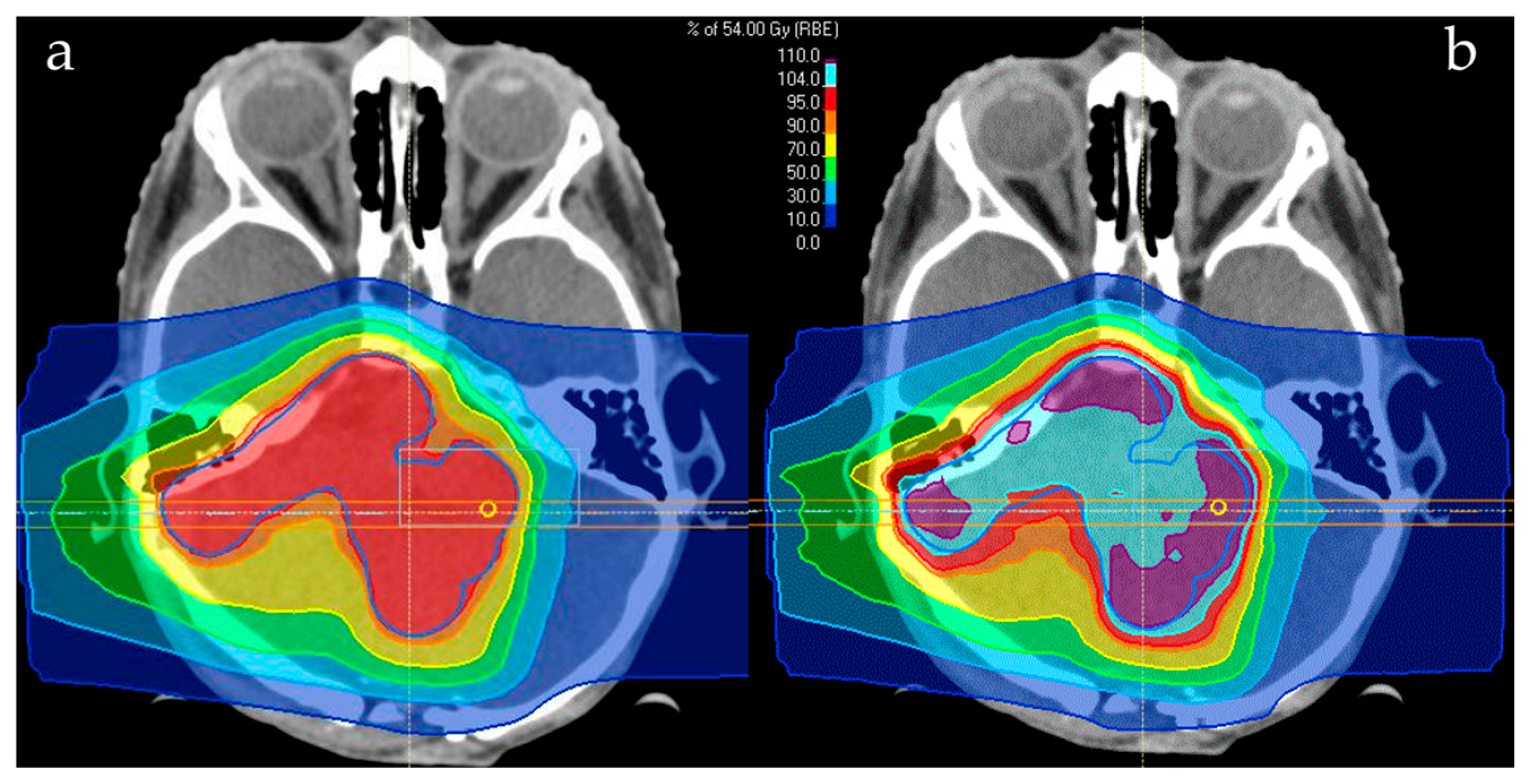
Representative comparison of biological dose distributions calculated using a fixed RBE of 1.1 (**a**) and the variable RBE model according to McNamara (**b**). The variable RBE model predicts an expansion of high biological dose regions (>90% of the prescribed dose), highlighting spatial dose heterogeneity compared with the conventional fixed RBE approach. The RICE lesion (small yellow contour) spatially overlaps with the region of increased biologically weighted dose predicted by the variable RBE model.

Application of all investigated variable RBE models, including Wedenberg, McNamara, and Carabe, resulted in higher biologically weighted dose estimates within RICE regions compared with calculations based on the conventional fixed RBE value of 1.1. Dose-volume histogram analyses demonstrated that variable RBE models predicted biologically relevant hotspots exceeding 100% of the prescribed reference dose within the target volume. The McNamara model showed the most pronounced hotspot formation and is shown in Figure 2 as an example.

**Figure 3:**
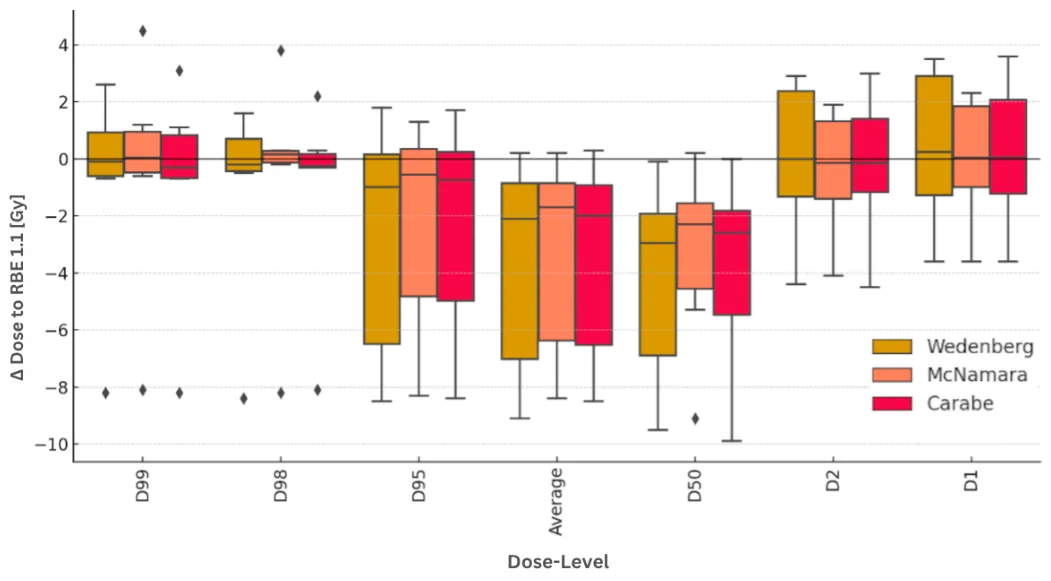
Relative dose differences (ΔDose) between the three variable RBE models (Wedenberg, McNamara, and Carabe) and the conventional fixed RBE model (RBE = 1.1) across predefined dose-volume metrics (D99–D1). ΔDose represents the difference between the RICE-to-PTV dose difference calculated using each variable RBE model and the corresponding difference calculated using the conventional fixed RBE of 1.1. Box plots illustrate the distribution of differences between RICE regions and the corresponding PTV in the six patients with RICEs. Symbols outside the whiskers indicate outliers.

Across all investigated variable RBE models, relative dose differences compared with the conventional fixed RBE model (RBE = 1.1) varied across the evaluated dose-volume metrics. The most pronounced differences were observed in the low- to intermediate-dose range, particularly between D95 and D50, whereas only minor differences were present at D99, D98, and in the high-dose region (D2 and D1). Among the investigated models, the Wedenberg model generally demonstrated the largest relative deviations from the fixed RBE approach at D95–D50. Model-based dose comparisons relative to the conventional fixed RBE model are summarized in Figure 3.

**Figure A1:**
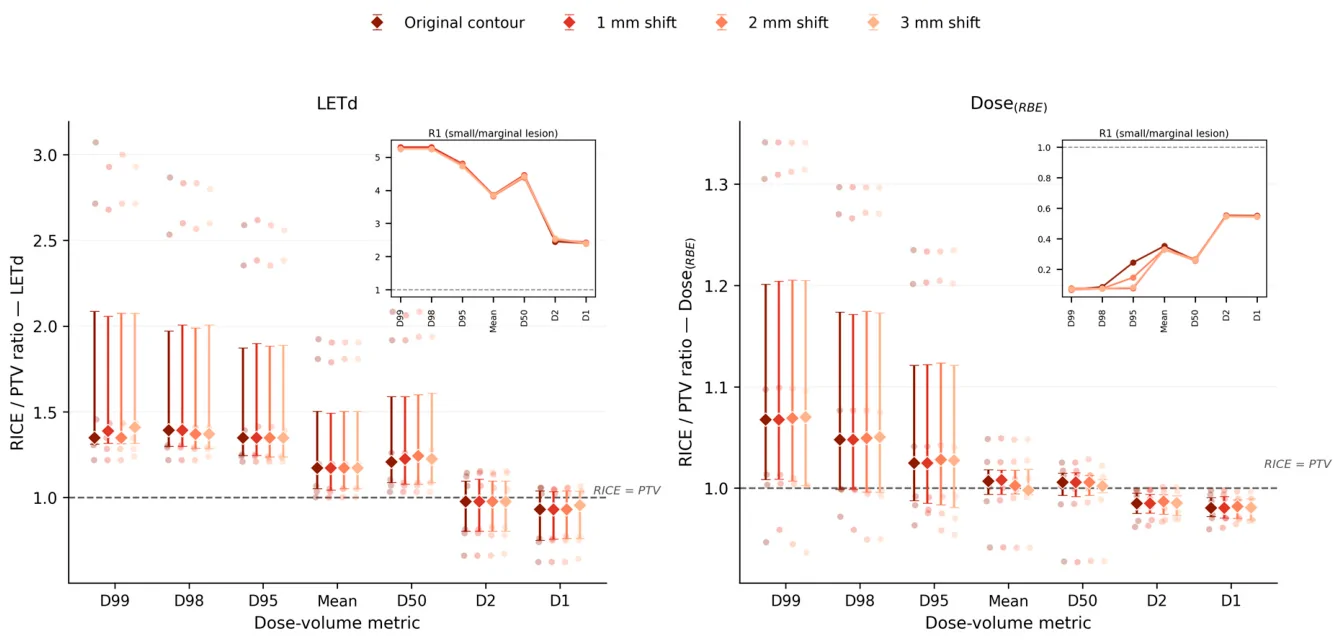
Margin sensitivity analysis of RICE to PTV dosimetric ratios under simulated contour shifts. RICE to PTV ratios for LETd (**left**) and Dose_(RBE)_ (**right**) across seven dose-volume levels (D99–D1), comparing the original RICE contour with contours shifted by 1, 2, and 3 mm in six directions. Diamonds and error bars represent the median and interquartile range across seven RICE regions; faint points show individual region-level ratios per shift direction. The dashed line indicates parity between RICE and PTV (ratio = 1). One RICE region, treated with single-field irradiation, is shown separately in the inset.

The robustness of these findings against potential contouring and registration uncertainty was confirmed by a margin sensitivity analysis, presented in Appendix C (Figure A1).

## 4. Discussion

The present study investigated the relationship between RICE and LET-associated biological dose after proton therapy in paediatric patients. The observed spatial association between RICE and elevated biologically weighted dose across all investigated variable RBE models supports the primary study hypothesis and extends previous observations from adult glioma cohorts to a paediatric population. The primary objective of this study was to investigate the spatial association between RICE and LET-associated biological dose following PRT in paediatric patients with low-grade glioma and ependymoma. Consistent with our hypothesis, RICE regions exhibited consistently higher biologically weighted dose metrics than the corresponding PTV across all investigated variable RBE models. Relative differences between the variable RBE models and the conventional fixed RBE approach were most pronounced in the low- to intermediate-dose range, whereas only minor deviations were observed in the highest-dose regions. Together, these findings support the concept that biologically weighted dose metrics identify spatial dose heterogeneity not fully represented by the conventional fixed RBE model.

**Figure A2:**
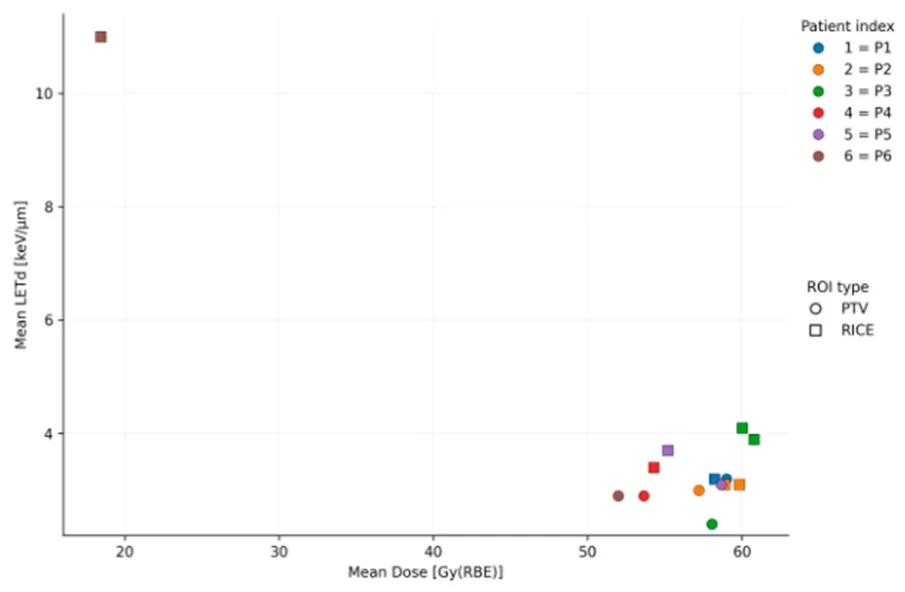
Two-dimensional distribution of ROI-level mean Dose_(RBE)_ and mean LETd in PTV and RICE regions. Mean Dose_(RBE)_ is displayed on the *x*-axis and mean LETd on the *y*-axis. Colours indicate patient identity (P1–P6), while marker shapes distinguish PTV regions (circles) from RICE lesions (squares). Corresponding patient and ROI identifiers are provided in Table A3.

Our observations are consistent with an increasing body of clinical evidence supporting the relevance of LET-informed biological dose evaluation for radiation-induced imaging changes after PRT. Previous voxel-based studies in adult glioma patients demonstrated an increased probability of RICE in regions characterized by elevated LET and variable RBE-weighted dose [12,13,18,21]. More recently, Bregman et al. developed the first voxel-based NTCP model for paediatric posterior fossa tumours and identified the combined dose–LET metric (D·LETd), younger age, and anatomical location within the pons as independent predictors of RICE [22]. Furthermore, RICE occurred predominantly in regions combining a high absorbed dose with intermediate-to-high D·LETd, reinforcing the biological relevance of LET-associated dose metrics in paediatric patients. A complementary two-dimensional visualization of mean Dose_(RBE)_ and LETd across RICE and PTV regions is provided in Appendix D (Figure A2), illustrating the heterogeneous Dose_(RBE)_–LETd characteristics of individual lesions. Although our study investigated a different tumour population and applied a region-based rather than voxel-wise analysis, the observed spatial association between RICE and biologically weighted dose is consistent with these findings. In contrast, the overall incidence of RICE in our cohort was substantially lower than that reported by Bregman et al. and also lower than rates described in adult low-grade glioma cohorts [9]. RICE was observed in 7.9% of the overall cohort and occurred exclusively in patients with ependymoma. This discrepancy is unlikely to be explained by differences in the radiological definition of RICE, as both studies adopted the definition previously proposed by our group [23], restricting RICE to newly occurring contrast-enhancing lesions outside the GTV to distinguish radiation-induced changes in normal brain tissue from intratumoural treatment-related imaging changes. Instead, differences in patient and treatment characteristics are the more likely explanation. Whereas our study exclusively included patients receiving focal cranial PRT, the cohort of Bregman et al. predominantly comprised patients with medulloblastoma, who almost invariably underwent craniospinal irradiation followed by a posterior fossa or tumour bed boost. Craniospinal irradiation exposes substantially larger volumes of the central nervous system than focal treatment alone and may therefore increase susceptibility to radiation-induced imaging changes. In addition, more than three quarters of patients in the Bregman cohort received chemotherapy, with approximately half undergoing concomitant treatment, which may have further contributed to the higher observed RICE incidence despite the inability to assess chemotherapy as an independent predictor because of the heterogeneity of treatment regimens. Age-related differences in tissue repair capacity, vascular vulnerability, and regenerative potential may further contribute to the lower incidence observed in paediatric focal proton therapy compared with adult low-grade glioma populations [24,25,26]. Nevertheless, the concordance of the spatial dosimetric findings despite these substantial differences in patient populations and treatment approaches suggests that the association between LET-associated biological dose and RICE represents a robust phenomenon across different PRT cohorts.

A central observation of the present analysis was that all investigated variable RBE models consistently identified higher biologically weighted dose estimates within RICE regions than within the corresponding PTV. Moreover, the largest deviations from the conventional RBE = 1.1 approach occurred in the low- to intermediate-dose range, whereas differences became progressively smaller towards the highest dose levels. This pattern suggests that biologically relevant dose heterogeneity may be particularly pronounced outside the high-dose core of the target volume, where physical dose distributions alone may underestimate biological effect [18,24,25,26,27]. Although the magnitude of the predicted dose differences varied between the Wedenberg, McNamara, and Carabe models, all demonstrated a comparable spatial pattern, supporting the robustness of the observed association despite differences in their underlying radiobiological assumptions.

These results contribute to the ongoing debate regarding the clinical relevance of variable RBE in PRT. The current standard of using a fixed RBE value of 1.1 provides a practical and reproducible basis for treatment planning [28,29]. However, it simplifies a complex biological process and does not account for spatial variations in LET, dose per fraction, or tissue-specific radiosensitivity. The present findings do not prove a causal relationship between LET, variable RBE, and RICE formation. Nevertheless, they provide clinical evidence that affected regions may coincide with areas of increased biological dose. This interpretation is further strengthened by the recent external validation of adult voxel-based RICE models by Palkowitsch et al. [30], who confirmed both increased periventricular sensitivity and the relevance of variable RBE for predicting radiation-induced imaging changes after PRT. Together, these studies support the view that evaluation of LET- and RBE-weighted dose complements conventional physical dose assessment, particularly for normal tissue risk characterization. At the same time, variable RBE models should be interpreted with appropriate caution. These models remain approximations of complex tissue responses, and differences between the Wedenberg, McNamara, and Carabe approaches reflect diverging assumptions regarding dose, LET, and tissue-specific parameters [23,31,32]. In small lesion volumes, model-based biological dose estimates may be sensitive to contouring uncertainty, image registration, and steep local dose gradients. Moreover, increased modelled biological dose does not necessarily imply clinically relevant toxicity. Many RICEs are asymptomatic and may regress spontaneously, suggesting that these lesions may represent transient blood–brain barrier disruption rather than irreversible parenchymal injury [33,34]. Variable RBE models should therefore currently be regarded as tools for risk exploration rather than definitive clinical decision criteria [13].

Exploratory NTCP estimates in patients with periventricular RICEs were generally low-to-moderate despite the occurrence of imaging changes. However, these results should be interpreted with caution, as the applied NTCP model was originally developed, trained, and calibrated in adult patients with low-grade glioma and was not intended for direct application to paediatric brain tumours. Paediatric patients may differ substantially from adults regarding neurodevelopment, tissue radiosensitivity, anatomical characteristics, and the spectrum and timing of radiation-induced late effects. The need for paediatric-specific normal-tissue models has been recognized by dedicated efforts such as the Paediatric Normal Tissue Effects in the Clinic (PENTEC) initiative, which addresses the limitations of extrapolating predominantly adult-derived normal-tissue data to childhood cancer populations. Consequently, the present analysis should be regarded as hypothesis-generating rather than a validation of the underlying risk model. Nevertheless, the observation that RICE occurred despite relatively low predicted complication probabilities suggests that risk estimates based primarily on physical dose and a fixed RBE of 1.1 may incompletely reflect biologically relevant tissue effects.

Our findings complement recent developments in biologically informed RICE modelling. Bauer et al. first demonstrated that incorporation of increased distal RBE and periventricular radiosensitivity into treatment planning could reduce the predicted risk of late MR-detected brain lesions in patients with low-grade glioma [35]. This concept was subsequently extended by Sallem et al., who proposed a model-based treatment planning strategy integrating biological risk estimates into proton plan optimization [20]. In parallel, Bregman et al. recently developed the first paediatric voxel-based NTCP model, demonstrating that the combined dose–LET metric (D·LETd), together with age and anatomical location, substantially improved prediction of RICE compared with absorbed dose alone [22]. Although these approaches differ in methodology and patient population, they consistently support the concept that incorporation of biological dose metrics improves characterization of radiation-induced normal tissue effects beyond conventional physical dose evaluation.

Against this background, our results provide complementary clinical imaging evidence demonstrating that RICE preferentially localized to regions characterized by increased LET-associated biological dose across multiple variable RBE models. Rather than advocating routine implementation of variable RBE-based treatment planning, our findings support continued development and prospective validation of biologically informed NTCP models and risk-adapted proton treatment planning strategies, particularly in paediatric patients, where even modest reductions in late toxicity may translate into substantial long-term clinical benefit. To confirm that these spatial associations were not attributable to image registration or contouring imprecision, we performed a margin sensitivity analysis with simulated contour shifts of up to 3 mm (Appendix C, Figure A1). This analysis demonstrated a robust, persistently elevated LET/RBE signature across all shift magnitudes, supporting the validity of our findings and minimizing the likelihood that they reflect contouring or alignment error rather than a genuine biological association. It should be noted that all observed RICE lesions were transient or self-limiting, with spontaneous regression in every case. Rather than diminishing the relevance of our findings, we believe this favourable clinical course supports the value of identifying and addressing LET/RBE-associated dose heterogeneity proactively during treatment planning, potentially preventing even mild, transient toxicity before it manifests clinically. This is particularly relevant in paediatric patients given their long life expectancy and cumulative treatment burden. Several limitations should be considered. The retrospective design and limited number of RICE events restricted statistical power and precluded multivariable modelling of clinical risk factors.

An additional limitation is the absence of spatially and dosimetrically matched non-RICE brain regions. The patient-specific PTV served as descriptive reference volumes and differed substantially from the small RICE lesions in size and spatial dose characteristics. Consequently, the observed dosimetric differences cannot establish whether LETd or variable RBE-weighted dose is independently associated with RICE localisation after accounting for anatomical location and conventional dose. Furthermore, the eight RICE regions originated from only six patients and should not be considered independent patient-level observations. Future studies incorporating appropriately matched non-RICE regions and accounting for patient-level clustering are needed to address this question.

Furthermore, the observed association between prescribed dose and RICE occurrence is univariable and may be confounded by tumour entity, as all RICE cases occurred in the ependymoma subgroup, which received significantly higher doses than the low-grade glioma subgroup. The additional descriptive comparison of oncological outcomes according to RICE status should likewise be interpreted with caution, as RICE was identified during follow-up and retrospective grouping may introduce immortal-time bias. Consequently, no conclusions regarding a prognostic association can be drawn. Given the retrospective design and the small number of observed RICE events, tumour-type-specific incidence estimates are subject to considerable statistical uncertainty, as reflected by the exact 95% confidence intervals (ependymoma: 14.0%, 95% CI 5.3–27.9%; low-grade glioma: 0%, 95% CI 0–10.6%). The overlap between the upper bound of the low-grade glioma interval and the lower bound of the ependymoma interval indicates that the retrospective cohort size limits the level of inference that can be drawn regarding a true absence of RICE in the low-grade glioma subgroup, and findings regarding tumour-type-specific risk should therefore be interpreted with caution and require confirmation in larger, ideally multicentre, cohorts. Nevertheless, the study was based on a prospectively maintained institutional registry comprising consecutive paediatric proton therapy patients and therefore reflects routine clinical practice. MRI examinations originated partly from external institutions, introducing some heterogeneity despite standardized review procedures. Furthermore, the use of retrospective image registration and lesion contouring may have introduced spatial uncertainty, particularly in small lesions located in regions with steep dose gradients; a dedicated margin sensitivity analysis (Appendix C, Figure A1) indicated that our findings were robust to this source of uncertainty. Finally, although three established variable RBE models were evaluated, all remain approximations of complex biological processes and should currently be regarded as exploratory tools for biological dose assessment rather than definitive predictors of clinical toxicity. Despite these limitations, this study has several important strengths. It represents one of the largest homogeneous paediatric cohorts of low-grade glioma and ependymoma treated exclusively with PRT, analysed using prospectively collected clinical data and systematic MRI follow-up. By combining volumetric lesion assessment with LET-based dosimetry and multiple variable RBE models, this study provides clinically relevant evidence supporting the spatial association between biologically weighted dose and RICE.

Future multicentre studies should validate these findings in larger paediatric cohorts and investigate whether integration of LET- and RBE-weighted dose metrics into NTCP modelling and biologically informed treatment planning can further improve individualized normal tissue risk assessment without compromising tumour control.

## 5. Conclusions

Proton therapy is a safe and effective treatment modality for paediatric patients with low-grade glioma and ependymoma, demonstrating excellent oncological outcomes and a low incidence of RICE. The observed spatial association between RICE and increased LET-associated biological dose across multiple variable RBE models suggests that biologically weighted dose metrics capture clinically relevant dose heterogeneity beyond the conventional fixed RBE approach. Together with emerging evidence from biologically informed risk modelling, these findings support continued refinement of LET- and RBE-informed NTCP models and proton treatment-planning strategies to further reduce normal tissue toxicity while maintaining excellent tumour control.

## Figures and Tables

**Table 1:** Comparison of Dose_(RBE)_ × LET products between RICE regions and PTV.

Dose Metric	RICE	PTV
D99	156.69	98.95
D98	162.09	104.09
D95	172.76	112.54
Mean dose	203.64	164.66
D50	196.32	156.64
D2	260.56	268.36
D1	265.09	286.47

**Table 2:** Comparison of LETd and Dose_(RBE)_ values between RICE regions and PTV. Values represent arithmetic means across eight individually delineated RICE regions and six patient-specific PTV.

	LETd	LETd	Dose_(RBE)_	Dose_(RBE)_
Dose Metric	RICE	PTV	RICE	PTV
D99	3.90	2.02	49.66	49.10
D98	3.96	2.07	50.06	50.26
D95	3.99	2.17	51.25	51.92
Mean dose	4.44	2.92	53.21	56.42
D50	4.45	2.75	52.66	56.89
D2	5.16	4.55	55.96	59.06
D1	5.26	4.83	56.06	59.35

## Data Availability

The original contributions presented in this study are included in the article. Further inquiries can be directed to the corresponding author.

## Abbreviations

The following abbreviations are used in this manuscript:

CI	Confidence Interval
Cm^3^	Cubic centimetre
Dose_(RBE)_	Relative-Biological-Effectiveness-weighted Dose
D·LETd	Dose–Linear Energy Transfer Metric
GTV	Gross Tumour Volume
GY	Gray
HIT	Heidelberg Ion-Beam Therapy Centre
LET	Linear Energy Transfer
LETd	Mean Dose Averaged Linear Energy Transfer
LGG	Low Grade Glioma
NTCP	Normal Tissue Complication Probability
PRT	Proton Beam Radio Therapy
PTV	Planning Target Volume
RBE	Relative Biological Effectiveness
RICE	Radiation-Induced Contrast Enhancement
ROI	Region of Interest
WHO	World Health Organization

## Appendix A

### Baseline Clinical and Radiotherapeutic Characteristics of the Radiological Cohort

**Table A1:** Baseline clinical and radiotherapeutic characteristics of the radiological cohort. Patient characteristics are presented for the overall cohort and stratified by tumour entity. Categorical variables are reported as *n* (%), and continuous variables as median (range), unless otherwise specified.

	Total (76)	LGG (33)	Ependymoma (43)
**Patients**			
male	43 [56.6%]	17 [51.5%]	26 [60.5%]
female	33 [43.4%]	16 [48.5%]	17 [39.5%]
**WHO-Classification**			
I	28 [36.8%]	28 [84.9%]	0 [0%]
II	8 [10.5%]	4 [12.1%]	4 [9.3%]
III	39 [51.3%]	0 [0%]	39 [90.7%]
unknown	1 [1.4%]	1 [3%]	0 [0%]
**Oncological Parameters**			
Progress	17 [22.4%]	5 [15.2%]	12 [27.9%]
Time to progress in months (median)	27	59	20
Overall survival	66 [86.8%]	29 [87.9%]	37 [86%]
Time of survival in months (median)	62.5	99	47
Follow-up time in months	38.5	48	34
**Radiobiological Parameters**			
median Dose [Gy]	54	54	59.4
radiation fractions (median)	30	30	30
**Age at Radiotherapy in years**			
median	7.2	11	5.6
minimum–maximum	1.1–17.9	1.6–17.8	1.1–17.9
**RICE**			
frequency of occurrence	6 [7.9%]	0	6 [14%]
time to RICE in months (median)	9.5	N/A	9.5
Average RICE volume in cm^3^	0.16 [0.01–0.68]	0	0.16 [0.01–0.68]

## Appendix B

### Variable RBE Model Formulations Implemented for Biological Dose Calculation

**Table A2:** D denotes the physical proton dose per fraction, and L denotes LETd. A photon reference α/β ratio of 2.0 Gy was assumed for brain tissue. The equations and numerical coefficients correspond to those implemented in the scripts used for the present analysis in RayStation version 11B.

Model	Reference	Implemented Formulation
Wedenberg	Wedenberg et al. (2013) [14]	$RBE_{dose}=\frac{1}{2\left(\sqrt{{\left(\frac{\alpha }{\beta }\right)}^{2}+4D\left(\frac{\alpha }{\beta }+0.434L\right)+4D^{2}}-\frac{\alpha }{\beta }\right)}$
McNamara	McNamara et al. (2015) [15]	$RBE_{dose}=\frac{1}{2\left(\sqrt{{\left(\frac{\alpha }{\beta }\right)}^{2}+4D\cdot \frac{\alpha }{\beta }\cdot 0.999064+\frac{0.35605}{\left(\frac{\alpha }{\beta }\right)L}+4D^{2}\cdot {\left(1.1012-0.0038703\sqrt{\left(\frac{\alpha }{\beta }\right)}\cdot L\right)}^{2}}-\frac{\alpha }{\beta }\right)}$
Carabe	Carabe et al. (2012) [16]	$RBE_{max}=0.843+0.154\left(\frac{2.686}{\frac{\alpha }{\beta }}\right)L$ $RBE_{min}=1.09+0.006\left(\frac{2.686}{\frac{\alpha }{\beta }}\right)L$ $RBE_{dose}=\frac{1}{2\left(\sqrt{{\left(\frac{\alpha }{\beta }\right)}^{2}+4D\cdot \frac{\alpha }{\beta }\cdot RBE_{max}+4\cdot RBE_{min}^{2}\cdot D^{2}}-\frac{\alpha }{\beta }\right)}$

## Appendix C

### Margin Sensitivity Analysis

To assess the robustness of the observed dosimetric associations against potential image registration and contouring uncertainty, which was particularly relevant given the small median RICE lesion volume of 0.16 cm^3^, a margin sensitivity analysis was performed. Each RICE contour was rigidly shifted by 1, 2, and 3 mm in six orthogonal directions (anterior, posterior, superior, inferior, left, right), and Dose_(RBE)_ and LETd were re-extracted at each of the seven predefined dose-volume levels (D99, D98, D95, mean, D50, D2, D1) for every shifted contour. For each shift magnitude, the median value across the six directions was calculated per lesion, and RICE-to-PTV ratios were computed for the original and all shifted contours across all eight evaluated RICE regions.

The contour-shift analysis demonstrated persistently elevated LETd ratios at D99–D50, whereas Dose_(RBE)_ ratios remained close to unity across the evaluated dose-volume levels. These findings suggest that the observed LETd differences were relatively insensitive to simulated contour displacements of up to 3 mm (Figure A1). The smallest, most marginally located lesion, which was treated with single-field irradiation, was displayed separately because of its distinct dosimetric profile and potential sensitivity to contouring and registration uncertainty.

## Appendix D

### Two-Dimensional Visualization of Dose_(RBE)_ and LETd in PTV and RICE Regions

To provide an additional two-dimensional visualization of the relationship between Dose_(RBE)_ and LET, ROI-level mean Dose_(RBE)_ and LETd values were extracted for all PTV and RICE regions of patients who developed RICE. In total, six PTV regions and eight RICE lesions from six patients were included. Each ROI is represented by a single point, with mean Dose_(RBE)_ plotted on the *x*-axis and mean LETd on the *y*-axis. Patient-specific colours and distinct marker shapes allow the assignment of RICE lesions to their corresponding PTVs. The analysis was intended as a descriptive visualization of the joint Dose_(RBE)_–LETd characteristics of the respective regions and does not represent a voxel-wise analysis.

The PTV values formed a relatively compact distribution, whereas RICE regions showed a broader range of LETd values. Seven of eight RICE lesions were located within a mean Dose_(RBE)_ range of approximately 54–61 Gy, with LETd values extending above those observed for most PTV regions. One RICE lesion showed a distinctly different pattern, characterized by markedly elevated LETd at a substantially lower mean Dose_(RBE)_. This lesion occurred in a patient treated using a single-field irradiation technique and therefore arose within a treatment geometry that differed from the remaining cases. The patient-specific assignment and corresponding ROI-level mean values are provided in Table A3.

**Table A3:** Patient-specific assignment of the six PTVs and eight RICE regions included in the two-dimensional dosimetric analysis. Anonymised patient and ROI identifiers allow the individual RICE lesions to be matched to their corresponding patient-specific PTVs in Figure A2. Patients P2 and P3 each contributed two separately delineated RICE regions. Values represent the region-level mean RBE-weighted dose and LETd used as coordinates in the scatter plot.

**Patient**	**ROI Type/Region**	**Dose_(RBE)_**	**LETd**
P1	PTV	59.00	3.2
P1	RICE	58.22	3.2
P2	PTV	57.21	3.0
P2	RICE1	58.89	3.1
P2	RICE2	59.83	3.1
P3	PTV	58.05	2.4
P3	RICE1	60.78	3.9
P3	RICE2	60.02	4.1
P4	PTV	53.63	2.9
P4	RICE	54.28	3.4
P5	PTV	58.65	3.1
P5	RICE	55.20	3.7
P6	PTV	51.99	2.9
P6	RICE	18.44	11.0
